# Modelling the cost-effectiveness of essential and advanced critical care for COVID-19 patients in Kenya

**DOI:** 10.1136/bmjgh-2021-007168

**Published:** 2021-12-06

**Authors:** Angela Kairu, Vincent Were, Lynda Isaaka, Ambrose Agweyu, Samuel Aketch, Edwine Barasa

**Affiliations:** 1 Health Economics Research Unit (HERU), KEMRI-Wellcome Trust Research Programme, Nairobi, Kenya; 2 Health Services Unit, KEMRI-Wellcome Trust Research Programme Nairobi, Nairobi, Kenya; 3 Epidemiology and Demography, KEMRI-Wellcome Trust Research Programme Nairobi, Nairobi, Kenya; 4 Centre for Tropical Medicine and Global Health, Nuffield Department of Medicine, University of Oxford, Oxford, UK

**Keywords:** COVID-19, health economics, health systems

## Abstract

**Background:**

Case management of symptomatic COVID-19 patients is a key health system intervention. The Kenyan government embarked to fill capacity gaps in essential and advanced critical care (ACC) needed for the management of severe and critical COVID-19. However, given scarce resources, gaps in both essential and ACC persist. This study assessed the cost-effectiveness of investments in essential and ACC to inform the prioritisation of investment decisions.

**Methods:**

We employed a decision tree model to assess the incremental cost-effectiveness of investment in essential care (EC) and investment in both essential and ACC (EC +ACC) compared with current healthcare provision capacity (status quo) for COVID-19 patients in Kenya. We used a health system perspective, and an inpatient care episode time horizon. Cost data were obtained from primary empirical analysis while outcomes data were obtained from epidemiological model estimates. We used univariate and probabilistic sensitivity analysis to assess the robustness of the results.

**Results:**

The status quo option is more costly and less effective compared with investment in EC and is thus dominated by the later. The incremental cost-effectiveness ratio of investment in essential and ACC (EC+ACC) was US$1378.21 per disability-adjusted life-year averted and hence not a cost-effective strategy when compared with Kenya’s cost-effectiveness threshold (US$908).

**Conclusion:**

When the criterion of cost-effectiveness is considered, and within the context of resource scarcity, Kenya will achieve better value for money if it prioritises investments in EC before investments in ACC. This information on cost-effectiveness will however need to be considered as part of a multicriteria decision-making framework that uses a range of criteria that reflect societal values of the Kenyan society.

**Key questions**

**What is already known?**

- The COVID-19 pandemic is responsible for substantial health effects in low-income and middle-income countries.
- The case management of COVID-19 is one of the key control interventions deployed by country health systems.
- Similar to other low-income and middle-income countries, Kenya had substantial gaps in both essential and advanced critical care at the beginning of the pandemic.

**What are the new findings?**

- Provision of essential care and advanced critical care for COVID-19 at the current health system capacity (status quo) was costly and the least effective strategy.
- Investment in both essential care and advanced critical care for COVID-19 is not cost-effective in Kenya when compared with investment in essential care.

**What do the new findings imply?**

- Prioritising investments in filling capacity gaps in essential care before investing in filling capacity gaps in advanced critical care for COVID-19 is more cost-effective in Kenya
- These findings are intended to inform the sequencing of investments in case management rather than the selection of either strategy, within a context of substantial resource constraint, and capacity gaps in both essential and advanced critical care or COVID-19.
- Kenya will need to consider these findings on cost-effectiveness within a multicriteria decision-making framework that use a range of criteria that reflect societal values.

## Introduction

The new COVID-19 has spread to nearly all countries and territories globally, with devastating impacts.^1^ As at 20 May 2021, 164.9 million cases of SARS-CoV-2 infections have been recorded resulting in 3.4 million deaths globally.^2^ Additionally, the excess deaths as recorded on 20 May 2021 were 7 596 818.^3^ In Kenya, 166 382 infections and 3035 deaths from COVID-19 have been recorded as of 20 May 2021,^2^ with some estimates suggesting that actual (observed and unobserved) deaths due to COVID-19 could be between 5 and 10 times the reported cases.^3^ Beyond direct health impacts, the COVID-19 pandemic is responsible for substantial indirect health effects that include the disruption of the delivery and access of routine health services. It is also responsible for negative socioeconomic impacts that include a slow-down of the global economy, increase in unemployment, impoverishment, disruption of schooling and threatening of food security, among others.^4^

The case management of COVID-19 is one of the key control interventions deployed by country health systems. COVID-19 is a highly contagious infectious disease transmitted by SARS-CoV-2 primarily via exposure to respiratory droplets.^1^ Clinically, COVID-19 presents as either of four severities namely (1) asymptomatic, (2) mild/moderate, (3) severe and (4) critical COVID-19.^5 6^ In Kenya, case management guidelines recommend asymptomatic and mild/moderate COVID-19 be managed at home (home based care), while patients with severe and critical COVID-19 are provided with institutional care in hospitals.^7 8^ Patients with severe COVID-19 are typically managed in general hospital wards, and receive essential care (EC) that may include supplemental oxygen support,^1^ whereas patients with critical COVID-19 are managed in intensive care units (ICUs) and provided with advanced critical care (ACC) such as mechanical ventilation, management of complications like respiratory failure, acute respiratory distress syndrome, thromboembolism, sepsis and septic shock, and multiorgan failure such as cardiac and acute kidney injury, provided in ICU.^1 9 10^

Like other low-income and middle-income countries, Kenya had substantial gaps in both essential and ACC at the beginning of the pandemic. For instance, it is estimated that only 58% of hospital beds had access to medical oxygen at the start of the pandemic.^11^ Further, only 16% of healthcare facilities in Kenya were able to monitor oxygen saturation and therapy through pulse oximetry, and the mean availability of tracer items for emergency breathing interventions (pulse oximeters, micronebuliser, beclomethasone and salbutamol inhalers, oxygen with tubing, flowmeter and humidifier, resuscitation bags, intubation devices with connecting tube, chest tubes with insertion sets and continuous positive airway pressure (CPAP) equipment) was only 13%.^11^ With regard to ACC, Kenya had only 540 ICU beds for a population of nearly 50 million, with only 22% of the population living within 2 hours of a facility with an ICU.^11^

Against this backdrop, the Kenyan government set out to invest in filling capacity gaps in both EC and ACC for COVID-19. However, because of resource constraints, gaps in both essential and advanced care persist 1 year into the pandemic.^12^ This has triggered discussions on the prioritisation of investments when resources are scarce. If, as it is evident, the Kenyan government is unable to fill capacity gaps in both essential and ACC, where should they start? In this paper, we carry out an economic evaluation to inform this decision. Specifically, we compare the cost-effectiveness of investments in EC and investments in advanced care in addition to EC to the current healthcare provision capacity (status quo) for the management of symptomatic COVID-19 patients with severe and critical disease in Kenya.

## Methods

### Study design

A decision tree analysis model in Tree Age Pro Healthcare 2020 was developed to evaluate the cost-effectiveness of investments in essential and ACC for the management of COVID-19 patients in Kenya from a health systems perspective. The model followed a cohort of 20 836 individuals, representative of all individuals hospitalised for COVID-19 illness (until 30 January 2021) through two treatment pathways, estimating costs and health gains. In both treatment pathways, the COVID-19 patients were diagnosed as either having severe or critical illness depending on the severity of symptoms as defined by the Kenya ministry of health COVID-19 case management guidelines.^7^ A diagnosis was followed by treatment of severe patients in a general ward and critical patients in the ICU. A time horizon of a patient care episode chosen.

### Model structure

**Figure 1:**
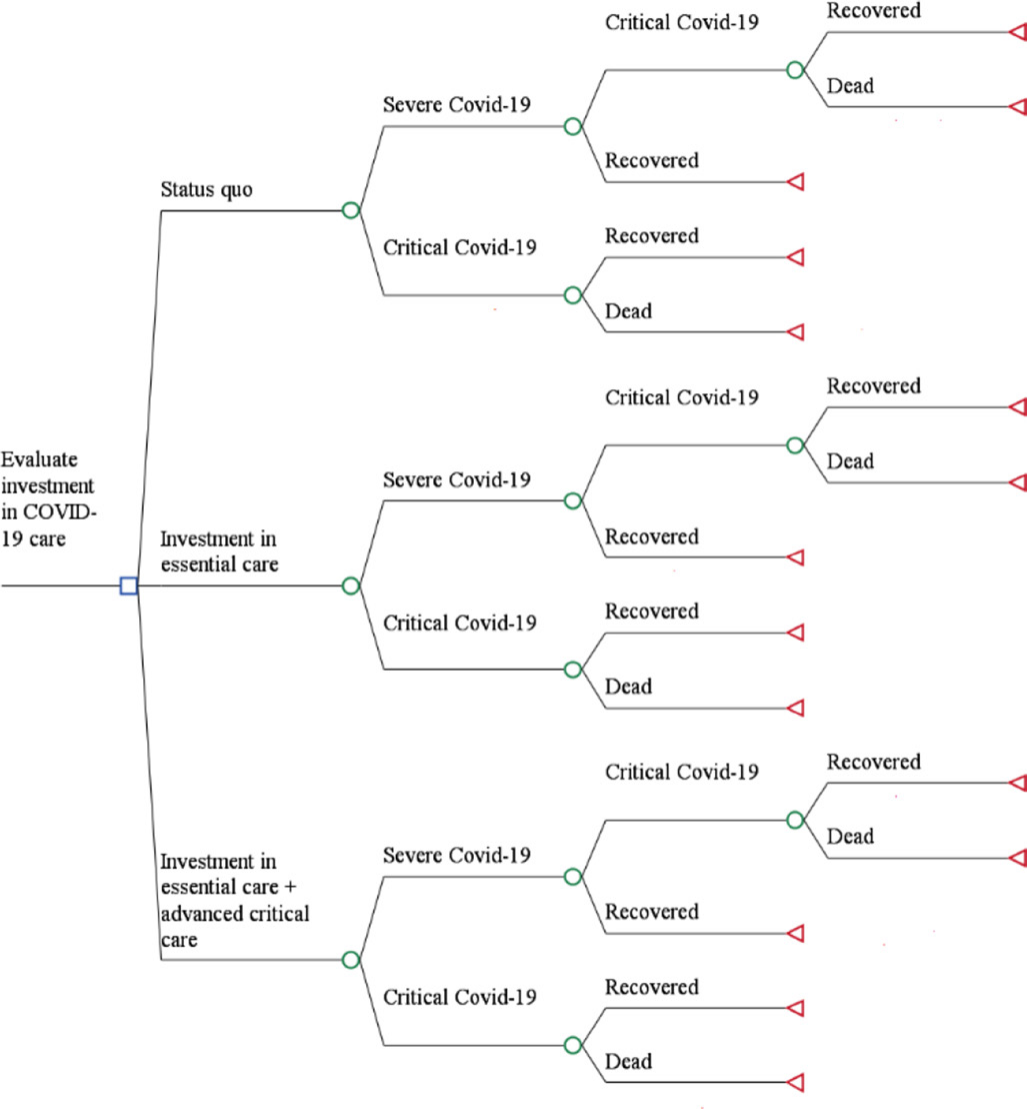
Schematic of decision tree mode.

Three different treatment strategies are compared (figure 1). Strategy 1 is defined as investment in filling EC gaps broadly comprising of supplementary oxygen therapy (when needed), administration of empiric antimicrobials, monitoring of vital signs and laboratory tests. Strategy 2 is defined as investment in ACC in addition to EC (EC+ACC). ACC encompasses management of patients in an ICU, advanced oxygen/ventilatory support, conservative fluid management, advanced organ monitoring and support, empiric antimicrobials and management of any complications.^7^ Strategy 3 is the baseline defined as status quo comprising of provision of EC and ACC within the current health system capacity. Online supplemental appendix 1 provides a detailed description of what is included in each intervention, which were defined based on expert consensus and the clinical guidelines implemented.^1 7^

10.1136/bmjgh-2021-007168.supp1 Supplementary data

The study population is hospitalised COVID-19 patients admitted between March 2020 and January 2021. The model assumes, in line with Kenya COVID-19 case management guidelines, that only patients with severe and critical disease are admitted in hospitals for inpatient care.^7^ Severe cases present with the following symptoms: fever or suspected respiratory infection, plus one of respiratory rate >30 breaths/min, severe respiratory distress or SpO2 <93%.^7^ Critical cases are those who meet any of the following criteria: respiratory failure requiring mechanical ventilation, shock and other organ failure requiring ICU care.^7^ All individuals completed the pathway when they were either recovered or dead. Data on the effectiveness of both comparators was obtained from current literature (see online supplemental file 1), and where unavailable assumptions were based on expert opinion (see online supplemental appendix 2). However, these data sources were limited by the regions/areas studied as the extent of disease outcomes varied globally. Key model input parameters are shown in table 1. In the cohort, COVID-19 patients were characterised as: (1) severe and (2) critical. These proportions were sourced from current literature.^13–15^

10.1136/bmjgh-2021-007168.supp2 Supplementary data

**Table 1:** Key cost-effectiveness model parameters

Parameter	Value (Lb; Ub)	Source
Population		
No of patients with severe or critical COVID-19 requiring hospitalisation in a year	20 836 (16 668; 25 003)	28
Proportion of hospitalised patients with severe COVID-19	0.86 (0.69; 1)	15
Proportion of hospitalised patients with critical COVID-19	0.14 (0.11; 0.17)	15
Proportion of severe COVID-19 that progress to critical (if essential critical care is provided)	0.0068 (0; 0.0068)	29
Proportion of severe COVID-19 that progress to critical (if essential critical care is not provided)	1	Author assumption
Proportion of severe COVID-19 that progresses to recovery (if essential critical care is provided)	0.99 (0.79; 1)	29
Proportion of severe COVID-19 that progresses to recovery (if essential critical care is not provided)	0	Author assumption
Proportion of critical COVID-19 that progresses to recovery (if advanced critical care is provided)	0.396 (0.316; 0.475)	23
Proportion of critical COVID-19 that progresses to recovery (if advanced critical care is not provided)	0	Author assumption
Health system capacity		
Proportion of baseline capacity for essential care	0.58	11
Proportion of baseline capacity for advanced critical care	0.22	11
Utilisation		
Length of hospital stay critical COVID-19 patients (days)	7 (4; 10)	23
Length of hospital stay for severe COVID-19 patients (days)	6 (3; 9)	30
Mortality rates		
Proportion of critical COVID-19 that progresses to death (if advanced critical care is provided)	0.604 (0.483; 0.724)	23
Proportion of critical COVID-19 that progresses to death (if advanced critical care is not provided)	1	Author assumption
DALYs		
Disability weight for critical care episode	0.655 (0.579; 0.727)	20
Disability weight for severe care episode	0.133 (0.088; 0.191)	19
Average age at death	55.5	14
Life expectancy	66.34	18
Unit costs		
Cost (US$) for critical care episode	599.91 (479.60; 719.41)	16
Cost (US$) for severe care episode	124.53 (99.62; 149.43)	16
Other		
Cost-effectiveness threshold per DALY averted	US$908.25	31 32

DALYs, disability-adjusted life-years; Lb, lower bound; Ub, upper bound.;

### Costing methods

An ingredients-based costing methodology was used to estimate unit costs of COVID-19 case management. The health system costs considered were associated with COVID-19 case management in hospitals (accommodation and overheads, staff, pharmaceuticals, non-pharmaceutical, personal protective equipment, oxygen therapy, ICU equipment, COVID-19 test, other laboratory tests and radiology tests). Details of the costing and results are reported elsewhere.^16^

### Effectiveness and cost-effectiveness measurement

The model’s primary outcome measure is the cost per disability adjusted life years (DALYs) averted. DALYs were calculated as the sum of years of life lost (YLL) and years of life with disability (YLD). We used standard methods to compute DALYs.^17^ DALYs were calculated using a discount rate of 3%, age weighting, Kenya’s life expectancy of 66.34,^18^ and assumed duration of illness of 12 days. The applied disability weight for severe respiratory infection was 0.133 (95% CI 0.088 to 0.190, 95% CI) from the Global Disease Burden study 2013^19^ for severe COVID-19 disease, and the disability weight of 0.655 (0.579–0.727) for ICU admission^20^ for critical COVID-19 disease.

The incremental cost-effectiveness ratio (ICER) was the measure of cost-effectiveness calculated as the net change in total costs and DALYs averted between providing essential services compared with proving critical care for COVID-19 cases.

ICER= $\frac{\left(C_{ec}-C_{cc}\right)}{\left(DALYs_{ec}-DALYs_{cc}\right)}$where the C**_ec_** is the total cost of EC for severe cases and C**_cc_** is the total of cost of critical care

The ICER was compared with the opportunity cost based Kenya cost-effectiveness threshold estimated by Woods *et al*^21^ and Ochalek *et al*^22^ which is US$908.

### Dealing with uncertainty

Sensitivity analysis was assessed using a one-way sensitivity analysis and a probabilistic sensitivity analysis (PSA). The one-way sensitivity analysis was conducted across all parameters to assess the effect of changes on the ICER. A 20% increase or decrease was implemented for parameters without confidence bounds. However, where possible, ranges for sensitivity analysis were based on upper and lower confidence intervals or IQR found within the systematic literature review (see online supplemental file 1). A separate one-way sensitivity analysis was also carried out on the assumption that 100% of individuals with severe COVID-19 that do not receive EC transition to critical care. In recognition that the author assumption may be extreme, we varied this assumption to 80% to explore its impact on the findings and conclusions. The PSA (Monte Carlo simulation) was performed to explore the effect of uncertainty across our model parameters. The key parameters included the per day costs for severe and critical patients, DALYs, length of stay, and the transition probabilities with defined distributions (online supplemental appendix 3). The analysis randomly sampled each parameter in our model simultaneously from their probability distribution and repeated this 1000 times to generate CIs around our estimates of cost per DALY averted. The CIs or variation of parameters and the effect on the cost-effectiveness were also evaluated.

Finally, the PSA was run to estimate the percentage change in parameters that would render EC and ACC cost-effective using the CET as the cut-off for this determination.

## Results

Table 2 shows the costs, DALYs and the ICER associated with the three analysis options. The findings show that investing to fill capacity gaps in both EC and ACC is the most costly option, followed by the status quo option. Investment to fill gaps in EC is the least costly option. Further, investments to fill capacity gaps in both EC and ACC is the most effective option (averts the most DALYs) while status quo option is the least effective option (averts the least DALYs). The status quo option is thus dominated by investment in EC since it is both more costly and less effective that the later. The ICER of investment in essential and ACC (EC +ACC) compared with investment in EC is US$1378.21 per DALY averted. This is higher than the cost-effectiveness threshold for Kenya (US$908), revealing that it is not cost-effective to prioritise the investment in ACC.

**Table 2:** Cost-effectiveness results (US$ 2020)

Strategy	Total costs (US$) (95% CI)	Total DALYs (95% CI)	Cost per DALY averted (US$)	Incremental cost per DALY averted (US$)
Essential care	16 197 611.92 (15 710 057.87 to 16 438 588.93)	22 508.76 (22 232.71 to 24 809.92)	719.61	
Status quo	17 474 037.20 (17 116 393.72 to 17 728 192.34)	77 621.36 (74 977.46 to 86 558.08)	225.11	−23.16
Essential care and advanced critical care	26 156 638.28 (25 594 910.55 to 26 468 628.66)	15 282.71 (15 027.06 to 16 817.24)	1711.52	1378.21

DALY, disability-adjusted life-year.

### Sensitivity analysis

The one-way sensitivity analysis on the assumption that 100% of severe COVID-19 patients transition to critical care are presented in table 3. When this assumption is varied to 80%, investing to fill capacity gaps in both EC and ACC is still the most costly option, followed by investing to fill capacity gaps in EC. The status quo option becomes the least costly option. The conclusion that investment to fill capacity gaps in both EC and ACC is not cost-effective is maintained.

**Table 3:** One-way sensitivity analysis (US$ 2020)

Strategy	Total costs (US$) (95% CI)	Total DALYs (95% CI)	Cost per DALY averted (US$)	Incremental cost per DALY averted (US$)
Status quo	15 428 195.90 (15 159 326.66 to 15 621 964.4)	66 493.97 (64 317.47 to 74 090.81)	232.02	
Essential care	16 197 611.92 (15 689 339.09 to 16 385 675.64)	22 508.76 (21 755.32 to 25 047.81)	719.61	17.49
Essential care and advanced critical care	26 156 638.28 (25 646 300.99 to 26 442 188.31)	15 282.71 (14 803.20 to 17 109.96)	1711.512	1378.21

DALY, disability-adjusted life year.

**Figure 2:**
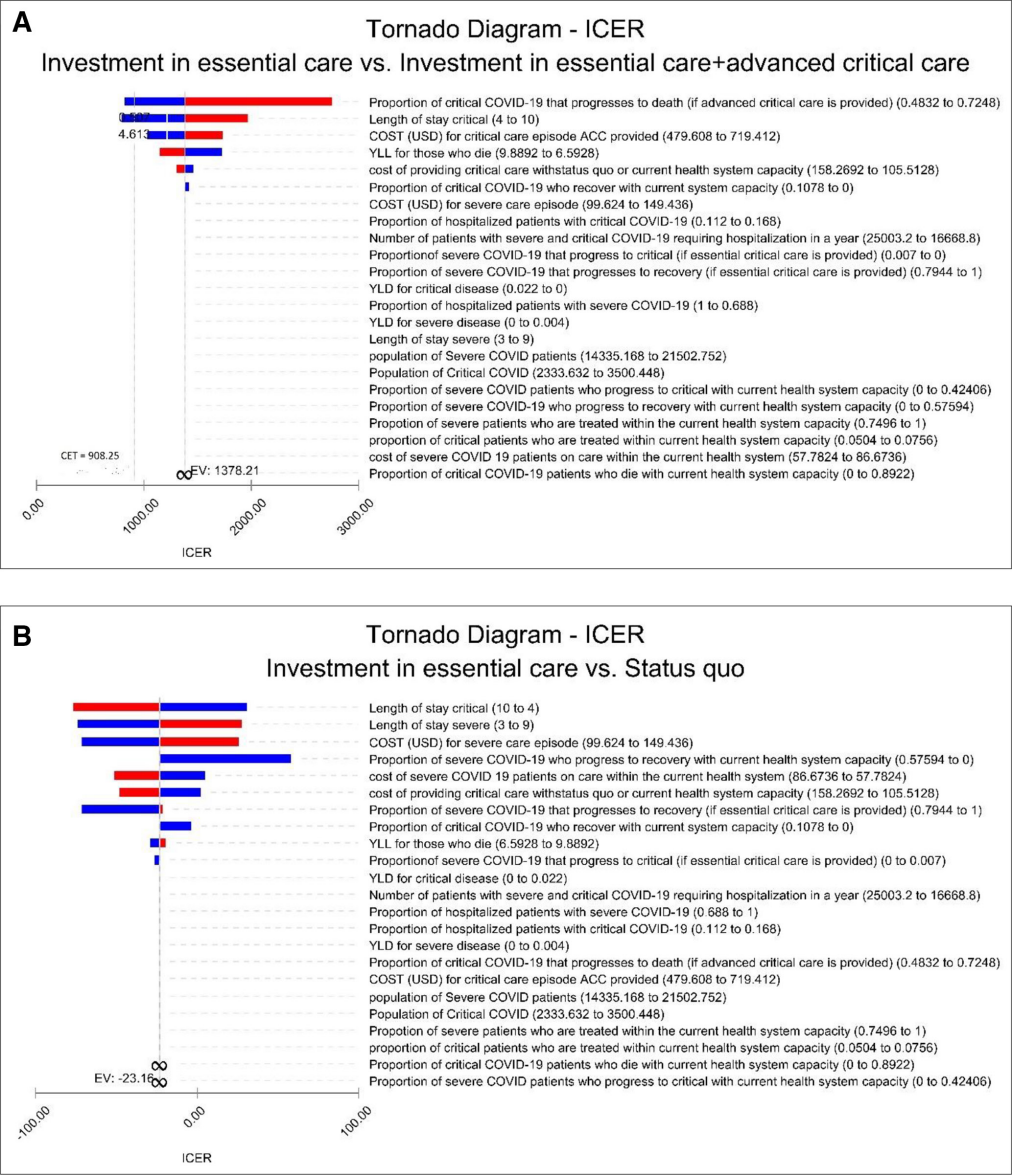
(A) Tornado diagram of univariate sensitivity analysis of the parameters affecting the ICER. (B) Tornado diagram of univariate sensitivity analysis of the parameters affecting the ICER. Red to blue colour represents a negative association between the parameter and the ICER. Blue to red colour represents a positive association between the parameter and the ICER. ACC, advanced critical care; ICER, incremental cost-effectiveness ratio; YLL, years of life lost; YLD, years of life lived with disability; EV, expected value.

Figure 2A summarises the results for the four main parameters that had the largest effect on the ICER. These are: (1) probability of critical COVID-19 patients to progress to death (ACC is provided) (lower mortality improves cost-effectiveness); (2) length of stay for critical COVID-19 patients (shorter length of stay improves cost-effectiveness); (3) the cost per day for ACC (less costly improves cost-effectiveness) and (4) years of life lost (if more lost life years can be averted, EC and ACC becomes more cost-effective).

These results indicate that if EC +ACC strategy was less costly (by unit cost reduction or the length of stay) or more effective (through targeting patients with more years of life to lose or reduced mortality), then this strategy would be more likely to be a cost-effective use of resources.

Figure 2B summarises the results for the main parameters that had the largest effect on the ICER comparing status quo to investment in EC. These are: (1) length of stay for critical COVID-19 patients; (2) length of stay for severe COVID-19 patients (both of which shorter length of stay improves cost-effectiveness); (3) the cost per day for EC (less costly improves cost-effectiveness) and (4) probability of severe COVID-19 patients to progress to recovery (current health system capacity) (higher recovery rates improves cost-effectiveness).

These results indicate that if status quo strategy was less costly (by unit cost reduction or the length of stay) or more effective (through less patients progressing to critical disease or more severe COVID-19 patients recovering), then this strategy would be more likely to be a cost-effective use of resources. Within the uncertainty range for the parameters reaching infinity, the incremental effectiveness passes through zero, which makes the ICER calculation undefined. Therefore, a bar would be invalid.

**Figure 3:**
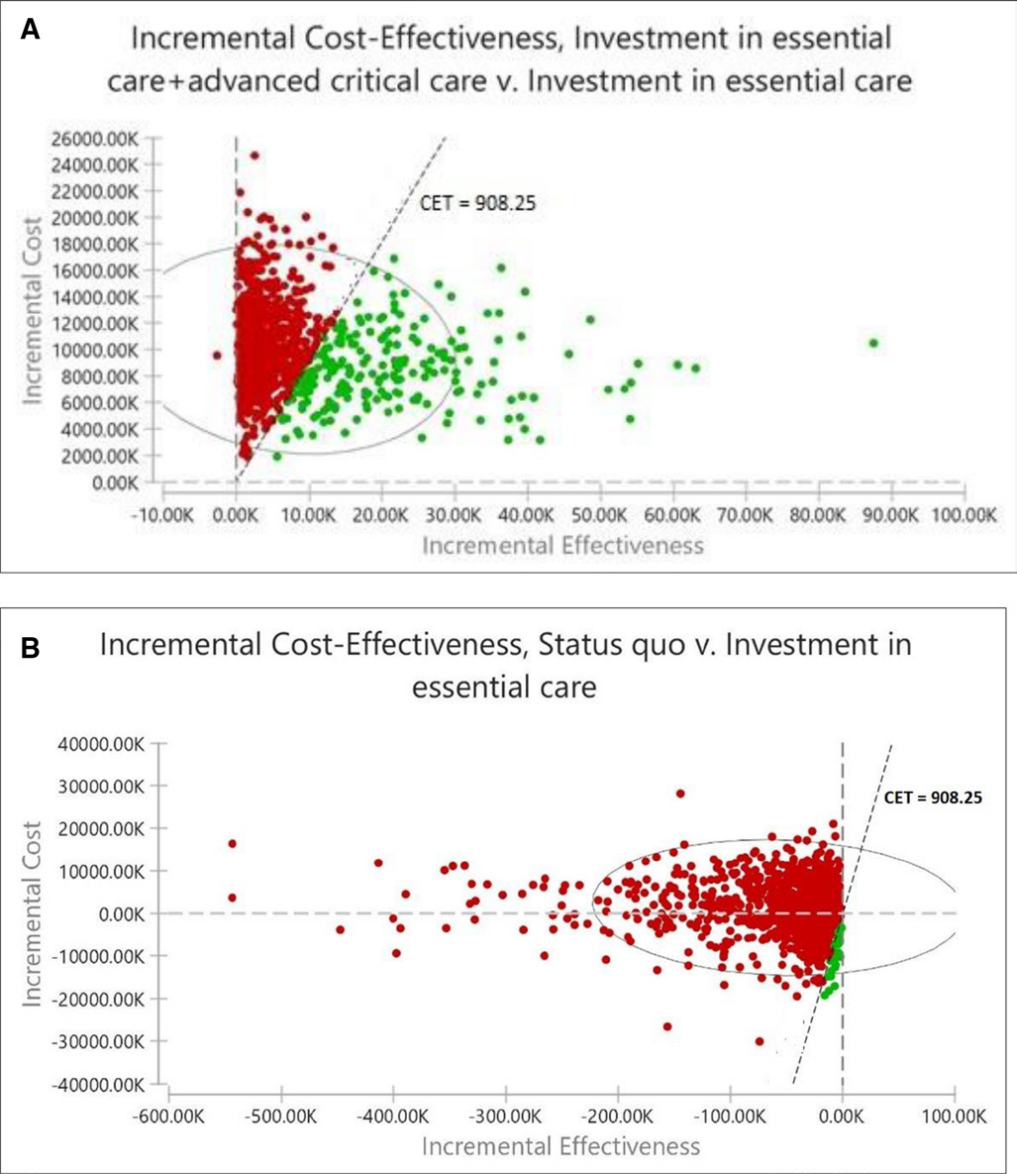
(A) Probabilistic sensitivity analysis of essential care versus essential and advanced critical care for COVID-19 patients. (B) Probabilistic sensitivity analysis of status quo versus essential care for COVID-19 patients. A dot represents a pair of values of incremental cost and incremental effectiveness. Green dots represent the points that are cost-effective (below the cost-effectiveness threshold (CET)). Red dots represent the points that are not cost-effective (above the CET).

Figure 3A–C outlines findings of the PSA. The region with green dots below the CET line shows all the points that are cost-effective. In figure 3A, the findings show that at a cost-effectiveness threshold of US$908.25, the probability of EC+ACC being the more cost-effective strategy is 22%. In figure 3B, the probability of status quo being the more cost-effective strategy is 2.7% when compared with EC, and 10.8% when compared with EC+AC strategy (figure 3C).

## Discussion

This study presents a cost-effectiveness analysis of investment strategies to fill gaps in Kenya’s health system capacity to provide case management for hospitalised COVID-19 patients. Specifically, we compare decisions to prioritise investments to fill existing gaps in EC versus prioritising to fill gaps in critical care in addition to EC for COVID-19 patients. Our findings show that it is more cost-effective to prioritise (ie, start with) investments to fill gaps in EC rather than fill gaps in ACC in addition to EC. We offer several reflections on these findings.

The first, what explains this finding? The status quo option is both less effective and more costly. While it is intuitive that the status quo option is less effective, the finding that it is more costly needs some explanation. Under the status quo option, a large proportion of patients that need essential critical care miss it, and hence transition to critical care, which is more costly. Despite investment in ACC in addition to EC averting more DALYs compared with investments in EC alone, it is substantially more expensive with cost per DALY averted being more than two times that of EC. Other than the obvious high cost of ACC, the proportion of hospitalised COVID-19 patients that need critical care (14%) is substantially lower than the proportion of patients that need EC (86%).^15^ Further, outcomes for ACC are poor with only 39.6% recovering.^23^ These factors combine to make an investment in critical care in addition to EC not cost-effective. These findings mirror a similar analysis carried out in South Africa that found purchasing additional ICU care during COVID-19 surges not cost-effective.^24^

Second, how should these findings be interpreted? We do not take these results to mean that Kenya should not invest in ACC. ACC is evidently a vital intervention in the management of COVID-19 disease as evidenced in this analysis where it averts additional DALYs when combined with EC, and may also have beneficial effects on the clinical management of other common conditions. However, within a context of (1) substantial gaps in both EC capacity and ACC capacity and (2) severe resource scarcity, what intervention should the Kenyan health sector prioritise? In other words, where should the Kenyan government start plugging the gaps? One option is to prioritise both, which within a budget constraint implies that both essential and ACC will remain suboptimal. This is indeed what we have observed in Kenya, with challenges in availability of EC that includes oxygen and critical care persisting 1 year since the onset of the pandemic despite arguably a low pandemic case burden compared with for instance countries in Europe and the USA.^2^ Our findings show that it is more cost-effective to start by prioritising investments in plugging gaps in EC before investing in plugging gaps in advanced care. These findings are, therefore, intended to inform the sequencing of investments in case management rather than the selection of either of essential or ACC. Suboptimal investment in both essential and critical care does not optimise health outcomes within a given budget.

Third, while the findings of this cost-effectiveness analysis are intended to inform priority setting for COVID-19 investments, they are to be considered within a multicriteria decision-making framework that reflect societal values. Our study findings provide quantitative evidence to inform such as multicriteria priority setting framework. While priority setting criteria that are based on ‘rule of rescue’ might favour investments in both essential and ACC, this is likely have high opportunity costs. A utilitarian consideration that aims to benefit the most people will favour the prioritisation of EC.

A key limitation to our analysis is the scarcity of local data to parametise the model. There is scant information on the clinical presentation, management and outcomes of COVID-19 patients in Africa. The duration of disability for DALYs is likely short (12 days) and should be adjusted when data becomes available. This, however, does not materially affect the study findings and conclusion since the contribution of disability to total DALYs is minimal as is often the case with acute conditions. We have, however, benefited from good-quality data on COVID-19 surveillance in Kenya that has bridged this data gap. This notwithstanding, we have used assumptions and estimates from other settings for some of the parameters. While this may affect the validity of the findings for the Kenyan setting, the sensitivity analysis reveals that the data are largely robust to variations in these parameters. Second, this analysis considers COVID-19 as an acute condition although there is emerging evidence of long-term effects^25–27^ and this has implications on the computation of DALYs. However, the information on long-terms effects is still evolving.

This study contributes to the growing body of literature on health economics analysis of COVID-19. Within the context of resource scarcity, Kenya will achieve better value for money if it prioritises investments in EC before investments in ACC. This information on cost-effectiveness will, however, need to consider alongside other priority setting considerations that are informed by the values of the Kenyan society.

## Data Availability

All data relevant to the study are included in the article or uploaded as online supplemental information. Not applicable.
