# Supplementary material for: Modelling the cost-effectiveness of essential and advanced critical care for COVID-19 patients in Kenya
Source: BMJ Glob Health. 2021 Dec 6;6(12):e007168. doi: 10.1136/bmjgh-2021-007168 (PMC8655343; doi:10.1136/bmjgh-2021-007168)
Supplement: Supplementary data [file bmjgh-2021-007168supp001.pdf]

APPENDICES

Appendix 1: Description of essential care and advanced critical care

Essential care	Advanced critical care
• Supplementary oxygen therapy (nasal cannula, Venturi mask, face mask with reservoir bag)<br>• Conservative fluid management (when necessary)<br>• Regular monitoring of vital signs (including pulse oximetry)<br>• Medication<br>- Empiric antimicrobials<br>- Administration of corticosteroids<br>• Radiology test (chest radiograph)<br>• Laboratory tests (SARS-CoV-2 RT-PCR test, full haemogram, urea electrolytes & creatinine (UECs), Aminotransferase alanine (ALT), Aspartate aminotransferase (AST), Alkanine phosphatase (ALP), Lactate dehydrogenase (LD), random blood sugar, C-reactive protein)	• Advanced oxygen/ventilatory support (mechanical ventilation)<br>• Conservative fluid management<br>• Cardiac and respiratory monitoring<br>• Parenteral nutrition (when indicated)<br>• Medication<br>- Empiric antimicrobials<br>Administration of corticosteroids<br>• Urine output monitoring<br>• Radiology test (CT scan (when indicated), chest radiograph)<br>• Laboratory tests (done routinely)<br>- Biochemistry (SARS-CoV-2 RT-PCR test, full haemogram, urea electrolytes & creatinine (UECs), liver function tests, random blood sugar, D-dimers, ferritin levels, C-reactive protein, blood gas analysis)<br>- Microbiology (blood culture, tracheal aspirate culture)<br>• Prevention of complications<br>- Pharmacological prophylaxis (anticoagulants)<br>- Regular repositioning of patients<br>• Management of complications<br>- Sepsis and septic shock<br>Antimicrobial therapy, fluid management, administration of vasopressors<br>Administration of blood and blood products (when indicated)<br>• Routine nursing care (nurse: patient ratio 1:1)<br>• Rehabilitation (physiotherapy)

Kairu A, *et al. BMJ Global Health* 2021; 6:e007168. doi: 10.1136/bmjgh-2021-007168

Appendix 2: Description of model assumptions and proportions calculations

The baseline capacity for essential care is 58% and that of advanced critical care is 22%. For the status quo strategy, the proportion of severe COVID-19 patients who receive essential care (EC) are 0.58 and a proportion of 0.0068 progress to critical disease. The model assumes that 100% of severe COVID-19 who do not receive EC progress to critical disease, of which a proportion of 0.22 receive advanced critical care (ACC) based on the current health system capacity. In addition, the costs of severe care episode and critical care episode have been multiplied the baseline capacity proportions. The status quo severe care episode cost is USD 72.23 (57.78; 86.67) and for critical care episode is USD 131.89 (105.51; 158.27).

For effectiveness, the years of life lived with disability (YLD) severe case is 0.004 (0; 0.004), YLD critical case is 0.022 (0; 0.022) and years of lives lost (YLL) is 8.241 (6.592; 9.889).

Appendix 3: Defined distributions for model parameters

Model parameters	Distribution
Total number and proportion of hospitalized patients	Normal
Transition probabilities	Beta
Costs	Gamma
Length of stay	Normal
DALYs	Lognormal

Kairu A, et al. BMJ Global Health 2021; 6:e007168. doi: 10.1136/bmjgh-2021-007168
