# Supplementary material for: Modelling the cost-effectiveness of essential and advanced critical care for COVID-19 patients in Kenya
Source: BMJ Glob Health. 2021 Dec 6;6(12):e007168. doi: 10.1136/bmjgh-2021-007168 (PMC8655343; doi:10.1136/bmjgh-2021-007168)
Supplement: Supplementary data [file bmjgh-2021-007168supp002.pdf]

Modelling the cost-effectiveness of essential and advanced critical care for COVID-19 patients in Kenya

Supplementary file 1

Parameter	Value (Lb; Ub)	Source
**Population**		
Number of patients with severe or critical COVID-19 requiring hospitalization in a year	20,836 (16,668; 25,003)	COVID-19 Transmission Dynamics Underlying Epidemic Waves in Kenya | medRxiv
Proportion of hospitalized patients with severe COVID-19	0.86	Clinical Characteristics and Outcomes of Patients Hospitalized for COVID-19 in Africa: Early Insights from the Democratic Republic of the Congo in: The American Journal of Tropical Medicine and Hygiene Volume 103 Issue 6 (2020) (ajtmh.org)
Proportion of hospitalized patients with critical COVID-19	0.14	Clinical Characteristics and Outcomes of Patients Hospitalized for COVID-19 in Africa: Early Insights from the Democratic Republic of the Congo in: The American Journal of Tropical Medicine and Hygiene Volume 103 Issue 6 (2020) (ajtmh.org)
Proportion of severe COVID-19 that progress to critical (*if essential critical care is provided*)	0.0068	Risk factors for developing into critical COVID-19 patients in Wuhan, China: A multicenter, retrospective, cohort study - EClinicalMedicine (thelancet.com)
Proportion of severe COVID-19 that progress to critical (*if essential critical care is NOT provided*)	1	Author assumption
Proportion of severe COVID-19 that progresses to recovery (*if essential critical care is provided*)	0.99	Risk factors for developing into critical COVID-19 patients in Wuhan, China: A multicenter, retrospective, cohort study - EClinicalMedicine (thelancet.com)
Proportion of severe COVID-19 that progresses	0	Author assumption

to recovery (*if essential critical care is NOT provided*)		
Proportion of critical COVID-19 that progresses to recovery (*if advanced critical care is provided*)	0.396	Epidemiology, outcomes, and utilization of intensive care unit resources for critically ill COVID-19 patients in Libya: A prospective multi-center cohort study (plos.org)
Proportion of critical COVID-19 that progresses to recovery (*if advanced critical care is NOT provided*)	0	Author assumption
**Health system capacity**		
Proportion of baseline capacity for essential care	0.58	Assessing the hospital surge capacity of the Kenyan health system in the face of the COVID-19 pandemic (plos.org)
Proportion of baseline capacity for advanced critical care	0.22	Assessing the hospital surge capacity of the Kenyan health system in the face of the COVID-19 pandemic (plos.org)
**Utilization**		
Length of hospital stay critical COVID-19 patients (days)	7 (4; 10)	Epidemiology, outcomes, and utilization of intensive care unit resources for critically ill COVID-19 patients in Libya: A prospective multi-center cohort study (plos.org)
Length of hospital stay for severe COVID-19 patients (days)	6 (3; 9)	Agweyu A. IL, Aman R., Kagucia E., Mwangangi M., Kasera K., Ng'ang'a W. Surveillance and epidemiologic evaluation of COVID-19 in Kenya (SEECK) protocol (unpublished). 2020.
**Mortality rates**		
Proportion of critical COVID-19 that progresses to death (*if advanced critical care is provided*)	0.604	Epidemiology, outcomes, and utilization of intensive care unit resources for critically ill COVID-19 patients in Libya: A prospective multi-center cohort study (plos.org)

Proportion of critical COVID-19 that progresses to death (*if advanced critical care is NOT provided*)	1	Author assumption
**DALYs**		
Disability weight for critical care episode	0.655 (0.579; 0.727)	Assessing disability weights based on the responses of 30,660 people from four European countries | Population Health Metrics | Full Text (biomedcentral.com)
Disability weight for severe care episode	0.133 (0.088; 0.191)	Disability weights for the Global Burden of Disease 2013 study - The Lancet Global Health
Average age at death	55.5	EPIDEMIOLOGICAL AND CLINICAL CHARACTERISTICS OF COVID-19 PATIENTS IN KENYA | medRxiv
Life expectancy	66.34	https://data.worldbank.org/indicator/SP.DYN.LE00.IN?locations=KE
**Unit costs**		
Cost (USD) for critical care episode	599.91	Examining unit costs for COVID-19 case management in Kenya | BMJ Global Health
Cost (USD) for severe care episode	124.53	Examining unit costs for COVID-19 case management in Kenya | BMJ Global Health
**Other**		
Cost-effectiveness threshold per DALY averted	0.5 times country's GDP per capita (USD 1,816.5)	What next after GDP-based... | Gates Open Research https://data.worldbank.org/indicator/NY.GDP.PCAP.CD?locations=KE
